# Evaluating the Clinical Significance of Pre- and Postoperative Platelet Function Testing in Coronary Artery Bypass Grafting Surgery

**DOI:** 10.3390/jcm15166440

**Published:** 2026-08-20

**Authors:** Milos Matkovic, Tina Novakovic, Aleksandar Milojevic, Vladimir Milicevic, Jelena Milin Lazovic, Vladimir Tutus, Filip Markovic, Nenad Lalovic, Milorad Bijelovic, Vuk Aleksic, Nemanja Aleksic

**Affiliations:** 1 Department of Cardiac Surgery, University Clinical Center of Serbia, 11000 Belgrade, Serbia; milojevic94a@gmail.com (A.M.); vladimir.milicevic.heartsurgery@gmail.com (V.M.); ner.vuk@hotmail.com (N.A.); 2 Faculty of Medicine, University of Belgrade, 11000 Belgrade, Serbia; 3 Department of Clinical Transfusion Medicine in Cardiovascular Surgery, University Clinical Center of Serbia, 11000 Belgrade, Serbia; tinkanova@gmail.com; 4 Institute for Medical Statistics and Informatics, Faculty of Medicine, University of Belgrade, 11000 Belgrade, Serbia; milinjelena@gmail.com; 5 Institute for Cardiovascular Diseases Dedinje, 11000 Belgrade, Serbia; vltutus@yahoo.com; 6 Clinic for Pulmology, University Clinical Center of Serbia, 11000 Belgrade, Serbia; flp.mark@gmail.com; 7 University Hospital Foca, 73300 Foca, Bosnia and Herzegovina; nenad.lalovic@gmail.com; 8 Faculty of Medicine Foca, University of East Sarajevo, 73300 Foca, Bosnia and Herzegovina; bijelovic@gmail.com (M.B.); aleksicvuk@hotmail.com (V.A.); 9 Thoracic Surgery Clinic, Institute for Pulmonary Diseases of Vojvodina, 21208 Sremska Kamenica, Serbia; 10 Clinical Hospital Center Zemun, 11000 Belgrade, Serbia

**Keywords:** CABG, platelet function testing, viscoelastic testing, bleeding, transfusion, patient blood management

## Abstract

**Background/Objectives**: Postoperative bleeding remains a major complication following coronary artery bypass grafting (CABG), contributing to transfusion requirements, reintervention, and morbidity. The objective of this study was to assess the clinical significance of preoperative platelet function testing and postoperative viscoelastic testing in patients undergoing elective isolated on-pump CABG. **Methods**: This prospective observational study included 708 patients undergoing surgery between January 2023 and January 2025. Preoperative platelet function was assessed with Multiplate^®^ impedance aggregometry (ADPHS and ASPI), and postoperative coagulation was assessed with ClotPro^®^ after cardiopulmonary bypass. The prespecified Multiplate thresholds were population- and assay-specific and were not intended as universal cutoffs. Postoperative bleeding was defined by cumulative chest-tube drainage during the first 24 h. **Results**: In unadjusted analyses, low ADPHS values (≤602.5 AU·min) were associated with higher platelet transfusion, cryoprecipitate use, and overall transfusion, and ADPHS showed modest discrimination for blood loss > 500 mL/24 h (AUC = 0.61, *p* = 0.041). Low ASPI values (≤453 AU·min) were associated with increased cryoprecipitate use and showed modest discrimination for blood loss > 1000 mL/24 h (AUC = 0.62, *p* = 0.005). After multivariable adjustment for baseline differences, neither ADPHS ≤ 602.5 (adjusted OR 1.01, 95% CI 0.74–1.39, *p* = 0.936) nor ASPI ≤ 453 (adjusted OR 0.89, 95% CI 0.64–1.23, *p* = 0.475) was independently associated with postoperative bleeding > 500 mL/24 h. Postoperative ClotPro^®^ parameters showed statistically significant associations with transfusion and bleeding, but their discriminatory performance was limited. **Conclusions**: Perioperative platelet function and viscoelastic test results were associated with bleeding and transfusion outcomes in unadjusted analyses, but discrimination was modest, and the platelet function cutoffs were not independently associated with bleeding > 500 mL after adjustment. These findings support a complementary hemostatic assessment role for combined POC testing but do not establish a standalone predictive model or an implementable transfusion algorithm.

## 1. Introduction

Postoperative bleeding remains one of the most critical complications following coronary artery bypass grafting (CABG). It contributes significantly to morbidity, mortality, and healthcare resource utilization. Data from the Society of Thoracic Surgeons (STS) database show that approximately 41% of patients require blood product transfusion postoperatively, while nearly 3% undergo reoperation for bleeding, underscoring the need for optimized perioperative management strategies [1].

Bleeding complications continue to be a major issue in cardiac surgery. Cardiopulmonary bypass (CPB) has detrimental effects on hemostasis and the coagulation cascade, contributing to increased intraoperative and postoperative blood loss, leading to increased morbidity and mortality [2,3]. Increased chest drain output also contributes to mortality through non-blood-transfusion-related mechanisms, leading to hypovolemia, anemia, hemodynamic instability, and cardiac tamponade [4]. This is exacerbated by the widespread use of potent antiplatelet agents such as ticagrelor, particularly in patients referred for coronary artery bypass grafting (CABG) following recent stenting and/or acute ACS, also with current guidelines suggesting continuation of aspirin throughout the perioperative period [5].

Because platelet function and recovery after single or dual antiplatelet therapy show substantial interindividual variability, point-of-care testing is increasingly used in daily cardiac surgical practice to identify patients at increased bleeding risk [6]. Impedance aggregometry (Multiplate^®^) provides a rapid quantification of platelet inhibition by aspirin, clopidogrel, and ticagrelor, while viscoelastic assays (ClotPro^®^, ROTEM^®^, TEG^®^) offer real-time assessment of global clotting dynamics and fibrinogen contribution to clot strength. Their use has been associated with improved patient blood management by reducing unnecessary transfusions, shortening ICU stays, and lowering reoperation rates, but evidence supporting the routine clinical use of POC tests remains limited to observational studies and small single-center randomized trials [7,8].

However, current European Association for Cardio-Thoracic Surgery (EACTS) guidelines only provide a Class IIb, Level B recommendation for integrating viscoelastic POC testing into perioperative algorithms, reflecting a paucity of large-scale prospective evidence. Consequently, utilization remains inconsistent across centers, and reimbursement is limited in many healthcare systems [9].

Our study aimed to assess the clinical significance of pre- and postoperative platelet function testing in CABG patients undergoing cardiopulmonary bypass.

## 2. Materials and Methods

### 2.1. Ethics Statement

This study was conducted in accordance with the Declaration of Helsinki. Before patient enrollment, the study was approved by the Departmental review board (IRB of the Clinic for Cardiac Surgery, University Clinical Centre of Serbia (approval No. 58, 10 January 2023). The study protocol and informed consent form were reviewed and approved, and written informed consent was obtained from all participants. Following the introduction of a centralized Ethics Committee for the University Clinical Centre of Serbia in 2026, the study underwent subsequent centralized ethical review and approval (No. 455/8, 7 May 2026) as part of the institutional reapproval of previously conducted studies.

### 2.2. Study Design

**Figure 1:**
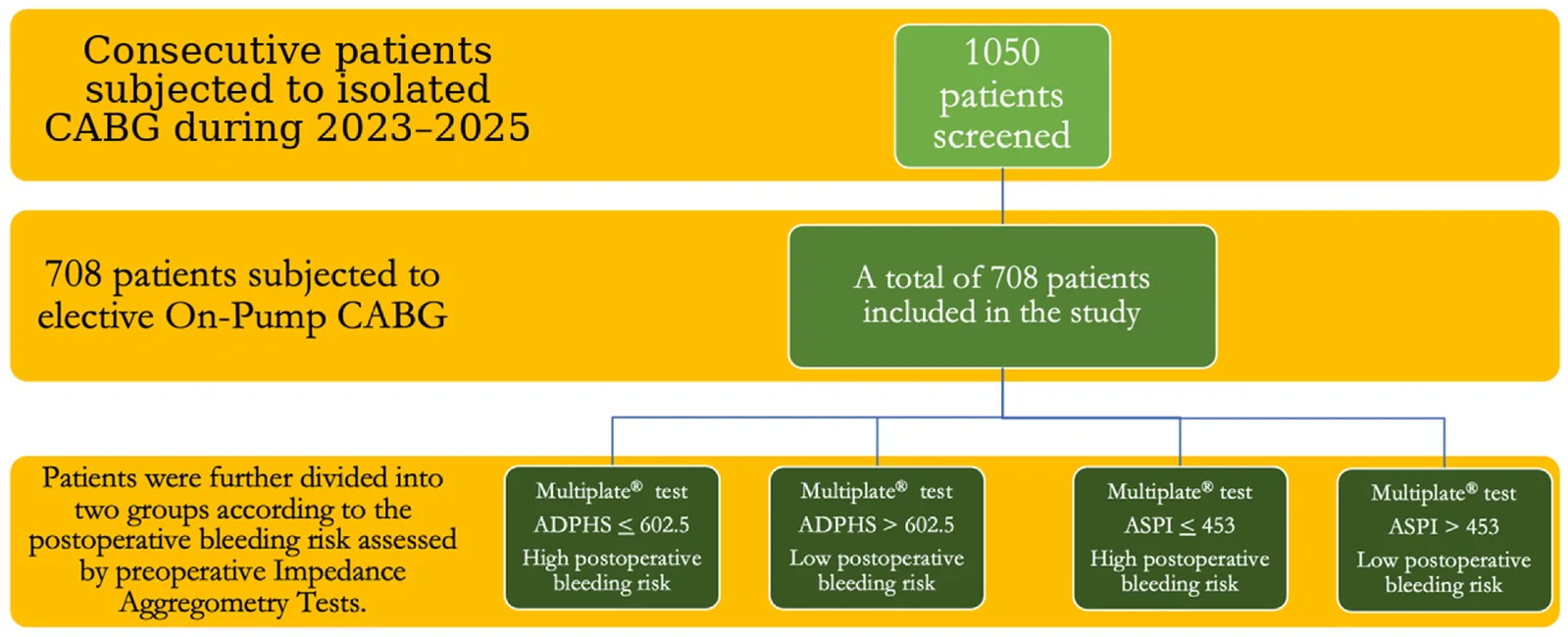
Study flowchart showing screening, inclusion, and stratification of patients into high- and low-risk bleeding groups by ADPHS and ASPI tests.

A prospective observational study was conducted. A total of 1050 consecutive patients undergoing first-time isolated CABG from January 2023 to January 2025 were screened. Patients undergoing off-pump CABG, minimally invasive CABG, combined procedures, or redo surgery were excluded. This resulted in 708 patients undergoing elective isolated on-pump CABG being included in the study. Patients were stratified according to preoperative impedance aggregometry results, as shown in Figure 1.

All patients underwent impedance aggregometry before surgery, and viscoelastic testing was performed after separation from CPB. Multiplate^®^ impedance aggregometry was used to assess platelet function in patients exposed to aspirin, clopidogrel, or ticagrelor. For the present study, ASPI > 453 AU·min and ADPHS > 602.5 AU·min were used as markers of lower bleeding risk. These cutoffs were derived previously in our institutional population [10] and are assay- and population-specific; they should not be interpreted as standardized universal or unit-independent thresholds. Postoperatively, ClotPro^®^ viscoelastic testing was used to assess coagulation status and to support blood-component transfusion decisions.

Patients classified as having a higher or lower bleeding risk by ASPI and ADPHS were compared with respect to erythrocyte and blood-component transfusion rates. In addition, postoperative viscoelastic parameters were evaluated in relation to bleeding and transfusion outcomes, including within the subgroups classified as higher bleeding risk by preoperative impedance aggregometry.

### 2.3. Study Outcomes and Definitions

This study’s outcomes included cumulative chest-tube drainage during the first 24 h; the occurrence of moderate and major postoperative bleeding; and perioperative exposure to erythrocytes, platelets, fresh frozen plasma, cryoprecipitate, and any blood-product transfusion.

Additional analyses evaluated the association of postoperative ClotPro^®^ EX-test and FIB-test parameters with bleeding and transfusion outcomes and their relationship with the preoperative Multiplate^®^ ADPHS and ASPI strata.

Postoperative bleeding was quantified as cumulative chest-tube drainage during the first 24 h after surgery. For the purposes of this study, moderate postoperative bleeding was defined as >500 mL within the first 24 h, and major postoperative bleeding as >1000 mL within the first 24 h.

Data were recorded preoperatively, intraoperatively, and postoperatively from patient records and the hospital electronic database. Demographic characteristics, comorbidities, operative characteristics, postoperative chest-tube output, erythrocyte and blood-component transfusions, need for reintervention, and ICU and hospital length of stay were recorded. Routine clinical laboratory data were collected as part of standard care; however, the additional multivariable models described below were restricted to baseline variables that differed significantly between the respective ADPHS and ASPI groups.

Preoperative characteristics were defined using standardized definitions, and the mortality risk was assessed using the EuroSCORE II. All clinically gathered data were systematically registered in accordance with standardized institutional protocols.

### 2.4. Surgical Procedure

All procedures were performed using cardiopulmonary bypass according to institutional protocols. Anesthesia was induced with midazolam (0.1–0.2 mg/kg), fentanyl (10–20 µg/kg), propofol (1–3 mg/kg), and rocuronium (0.6 mg/kg) or cisatracurium (0.15 mg/kg), and was maintained with sevoflurane (0.7–1.0 MAC) and remifentanil infusion (10–20 µg/kg/h). The CPB circuit was primed with 1100 mL Ringer’s solution, 15 g mannitol, 1 g tranexamic acid, and 5000 IU heparin. Standard aortic cannulation and two-stage venous cannulation were used. Myocardial protection was achieved with cold crystalloid cardioplegia. Left and right internal mammary arteries, the radial artery, and great saphenous vein grafts were used as appropriate. A cell-saver system was used for blood salvage and reinfusion in all patients.

### 2.5. Transfusion Strategy and Availability of POC Results

Perioperative transfusion decisions followed the institutional patient blood management protocol. In hemodynamically stable patients, erythrocyte transfusion was considered at a hemoglobin concentration < 7 g/dL; in hemodynamically unstable patients, a threshold of <8 g/dL was used. Platelet, fresh frozen plasma, and cryoprecipitate administration was guided by point-of-care test results together with clinical assessment rather than by a single fixed numerical cutoff. Preoperative Multiplate^®^ results were available to the treating clinicians and could inform platelet-component therapy. Post-CPB ClotPro^®^ results were also available and were used to guide fresh frozen plasma and cryoprecipitate administration. Thus, POC test results were not blinded to clinicians involved in transfusion decisions.

### 2.6. Blood Sampling and POC Tests

Venous blood sampling was performed at predefined perioperative time points: (1) preoperatively, within 24 h before surgery; (2) immediately after protamine administration following separation from CPB; and (3) postoperatively in the ICU when clinically indicated during the first 6 h after admission. These time points were selected to characterize baseline platelet function, early post-CPB coagulation changes after reversal of heparinization, and early postoperative hemostatic recovery. Samples were collected using atraumatic venipuncture or indwelling central venous lines, depending on the clinical setting.

For preoperative platelet function assessment, whole blood was drawn into 3 mL hirudin-anticoagulated tubes (Roche Diagnostics, Mannheim, Germany) and processed within 2 h of collection. Platelet function was evaluated using the Multiplate^®^ impedance aggregometry system (Roche Diagnostics GmbH, Mannheim, Germany). The ASPI test (arachidonic acid-induced platelet aggregation) was used to assess aspirin-related inhibition, whereas the ADPHS test (adenosine diphosphate-induced platelet aggregation) was used to evaluate P2Y12-receptor inhibition from clopidogrel or ticagrelor. The study thresholds, ADPHS ≤ 602.5 AU·min and ASPI ≤ 453 AU·min, were derived previously in our patient population [10]. They are specific to the Multiplate assay and the studied population and are not proposed as standardized, universal, or unit-independent thresholds.

Post-CPB coagulation status was assessed with the ClotPro^®^ viscoelastic hemostatic analyzer (Enicor GmbH, Munich, Germany). Whole blood was collected in 3.2% sodium-citrate tubes and tested within 30 min of sampling. The EX-test was performed to assess the extrinsic coagulation pathway, with clot formation time (CFT) and amplitude at 10 min (A10) as primary variables. The FIB-test, performed in parallel, was used to assess the fibrinogen contribution to clot strength, with FIB-test A10 as the main parameter.

Postoperative ICU blood sampling, when clinically indicated, followed the same protocol. This allowed evaluation of dynamic changes in platelet function and clot strength during the early recovery period. All samples were analyzed immediately by trained laboratory personnel in accordance with the manufacturer’s instructions. Quality control procedures were performed daily to ensure analytical reliability. Assay results were documented prospectively and used for correlation with perioperative bleeding, transfusion requirements, and postoperative outcomes.

### 2.7. Statistical Analysis

Descriptive statistics were calculated for baseline demographic and clinical characteristics and treatment outcomes. Distributional normality was assessed using histograms, Q-Q plots, and the Shapiro–Wilk test. Continuous variables are presented as mean ± standard deviation for normally distributed data or median with 25th–75th percentiles for non-normally distributed data. Categorical variables are presented as counts and percentages. Between-group differences were analyzed using Student’s *t*-test, the Mann-Whitney U test, or Pearson’s chi-squared test, as appropriate. Receiver-operating characteristic (ROC) analysis was used to evaluate the discriminatory performance of ADPHS, ASPI, and ClotPro^®^ parameters for postoperative bleeding and transfusion outcomes. For the additional descriptive analysis of absolute blood loss and transfused blood-component quantities requested during peer review, continuous ADPHS data were analyzed on an available-case basis of imputation.

To account for potential confounding from baseline differences, additional multivariable logistic regression analyses were performed with postoperative bleeding > 500 mL within the first 24 h as the dependent variable. The ADPHS model was adjusted for sex, hyperlipidemia, diabetes mellitus, and ejection fraction, whereas the ASPI model was adjusted for age and diabetes mellitus. Covariates were selected from baseline variables that differed significantly between the respective groups. Adjusted odds ratios (ORs), 95% confidence intervals (CIs), and *p*-values were reported.

For ROC analyses, 95% confidence intervals were calculated for AUC estimates. AUC values close to 0.50 were interpreted as poor discrimination. For parameters in which lower values were associated with greater bleeding or transfusion risk, AUC values below 0.50 were interpreted as reflecting an inverse direction of association rather than clinically useful standalone predictive performance. Statistical analyses were performed using IBM SPSS Statistics for Windows, version 21.0 (Armonk, NY, USA).

## 3. Results

### 3.1. Patients

A total of 708 patients undergoing isolated elective on-pump CABG were included in the study, further divided into ADPHS and ASPI groups with respect to the cutoff values. Detailed baseline characteristics are presented in Table 1.

**Table 1:** Baseline characteristics and perioperative data according to ADPHS and ASPI cutoff values.

	ADPHS ≤ 602.5 (n = 246)	ADPHS > 602.5 (n = 462)	*p*-Value	ASPI ≤ 453 (n = 224)	ASPI > 453 (n = 484)	*p*-Value
**Demographics and** **Risk Factors**						
**Age (years), mean ± SD**	65.9 ± 8.0	64.9 ± 8.2	0.136	66.3 ± 7.6	64.8 ± 8.3	0.017
**Male sex, n (%)**	201 (81.7)	334 (73.7)	0.017	165 (73.7)	377 (78.2)	0.182
**Female sex, n (%)**	45 (18.3)	119 (26.3)	0.017	59 (26.3)	105 (21.8)	0.182
**Hypertension, n (%)**	238 (96.7)	435 (96.0)	0.630	218 (97.3)	462 (95.9)	0.334
**Hyperlipidemia, n (%)**	197 (80.1)	390 (86.1)	0.039	186 (83.0)	407 (84.4)	0.636
**COPD, n (%)**	27 (11.0)	35 (7.7)	0.149	17 (7.6)	46 (9.5)	0.397
**Prior stroke/TIA, n (%)**	6 (2.4)	22 (4.9)	0.120	9 (4.0)	20 (4.1)	0.935
**Diabetes mellitus, n (%)**	91 (37.0)	205 (45.3)	0.035	77 (34.4)	221 (45.9)	0.004
**Peripheral vascular disease, n (%)**	5 (2.0)	8 (1.8)	0.803	2 (0.9)	11 (2.3)	0.201
**Smoking, n (%)**	53 (21.5)	115 (25.4)	0.256	47 (21.0)	123 (25.5)	0.189
**PCI > 90 days, n (%)**	30 (12.2)	68 (15.0)	0.306	33 (14.7)	65 (13.5)	0.656
**Previous MI, n (%)**	51 (20.7)	74 (16.3)	0.147	42 (18.8)	86 (17.8)	0.771
**MI > 90 days, n (%)**	86 (35.0)	148 (32.7)	0.540	65 (29.0)	170 (35.3)	0.101
**Perioperative Data**						
**Ejection fraction (%)**	59.8 ± 26.2	56.6 ± 26.4	0.015	58.8 ± 26.8	57.9 ± 26.4	0.609
**CPB time (min)**	56.0 ± 34.1	57.2 ± 37.3	0.671	58.9 ± 36.2	55.8 ± 36.3	0.289
**Aortic cross-clamp (min)**	31.7 ± 20.9	32.4 ± 21.3	0.692	33.6 ± 21.8	31.4 ± 20.9	0.217
**ICU stay (days)**	2.9 ± 2.5	3.3 ± 2.7	0.060	3.1 ± 2.1	3.2 ± 2.8	0.798
**Ventilation time (h)**	3.5 ± 1.8	3.5 ± 2.0	0.953	3.4 ± 1.5	3.6 ± 2.2	0.101

Perioperative antithrombotic therapy is summarized in Table 2 Preoperatively, aspirin was recorded in 632/708 patients (89.3%), clopidogrel in 340/708 (48.0%), and ticagrelor in 85/708 (12.0%). Anticoagulant exposure included LMWH in 329/708 (46.5%), vitamin K antagonists in 11/708 (1.6%), and NOACs in 13/708 (1.8%). Postoperatively, aspirin was recorded in 690/708 (97.5%), clopidogrel in 229/708 (32.3%), ticagrelor in 43/707 (6.1%), LMWH in 469/707 (66.3%), UFH in 3/708 (0.4%), vitamin K antagonists in 37/708 (5.2%), and NOACs in 18/708 (2.5%). These categories were not mutually exclusive.

**Table 2:** Perioperative antithrombotic therapy in the study cohort.

Therapy	Preoperative	Postoperative
Aspirin	632/708 (89.3%)	690/708 (97.5%)
Clopidogrel	340/708 (48.0%)	229/708 (32.3%)
Ticagrelor	85/708 (12.0%)	43/707 (6.1%)
LMWH	329/708 (46.5%)	469/707 (66.3%)
UFH	0/708 (0.0%)	3/708 (0.4%)
Vitamin K antagonist	11/708 (1.6%)	37/708 (5.2%)
NOAC	13/708 (1.8%)	18/708 (2.5%)

Values are n/N (%). Medication categories were not mutually exclusive. One postoperative observation was missing for ticagrelor and LMWH.

Among the 708 patients included, 246 (34.7%) had ADPHS ≤ 602.5 and 462 (65.3%) had ADPHS > 602.5. The mean age was approximately 65 years, with no significant difference between the groups. Male sex was more frequent in the low-ADPHS group (81.7% vs. 73.7%, *p* = 0.017). Hyperlipidemia (80.1% vs. 86.1%, *p* = 0.039) and diabetes mellitus (37.0% vs. 45.3%, *p* = 0.035) were more common in patients with ADPHS > 602.5, and ejection fraction also differed between groups (Table 1). Other reported comorbidities and perioperative parameters were broadly comparable. Mean EuroSCORE II was 2.1 ± 1.2 in the ADPHS ≤ 602.5 group and 2.3 ± 1.4 in the ADPHS > 602.5 group.

When patients were stratified according to ASPI values, 224 (31.6%) had ASPI ≤ 453 AU·min and 484 (68.4%) had ASPI > 453 AU·min. Patients in the low-ASPI group were older (66.3 ± 7.6 vs. 64.8 ± 8.3 years, *p* = 0.017). Diabetes mellitus differed between the groups (34.4% vs. 45.9%, *p* = 0.004), whereas the other reported demographic, cardiac-history, and perioperative variables were broadly comparable (Table 1).

### 3.2. Preoperative Platelet Function Testing

Preoperative platelet function was assessed using Multiplate^®^ impedance aggregometry. Patients were stratified according to the prespecified, population-specific ADPHS and ASPI thresholds (≤602.5 AU·min and ≤453 AU·min, respectively). These cutoffs were used for within-study stratification and are not presented as universal thresholds.

**Figure 2:**
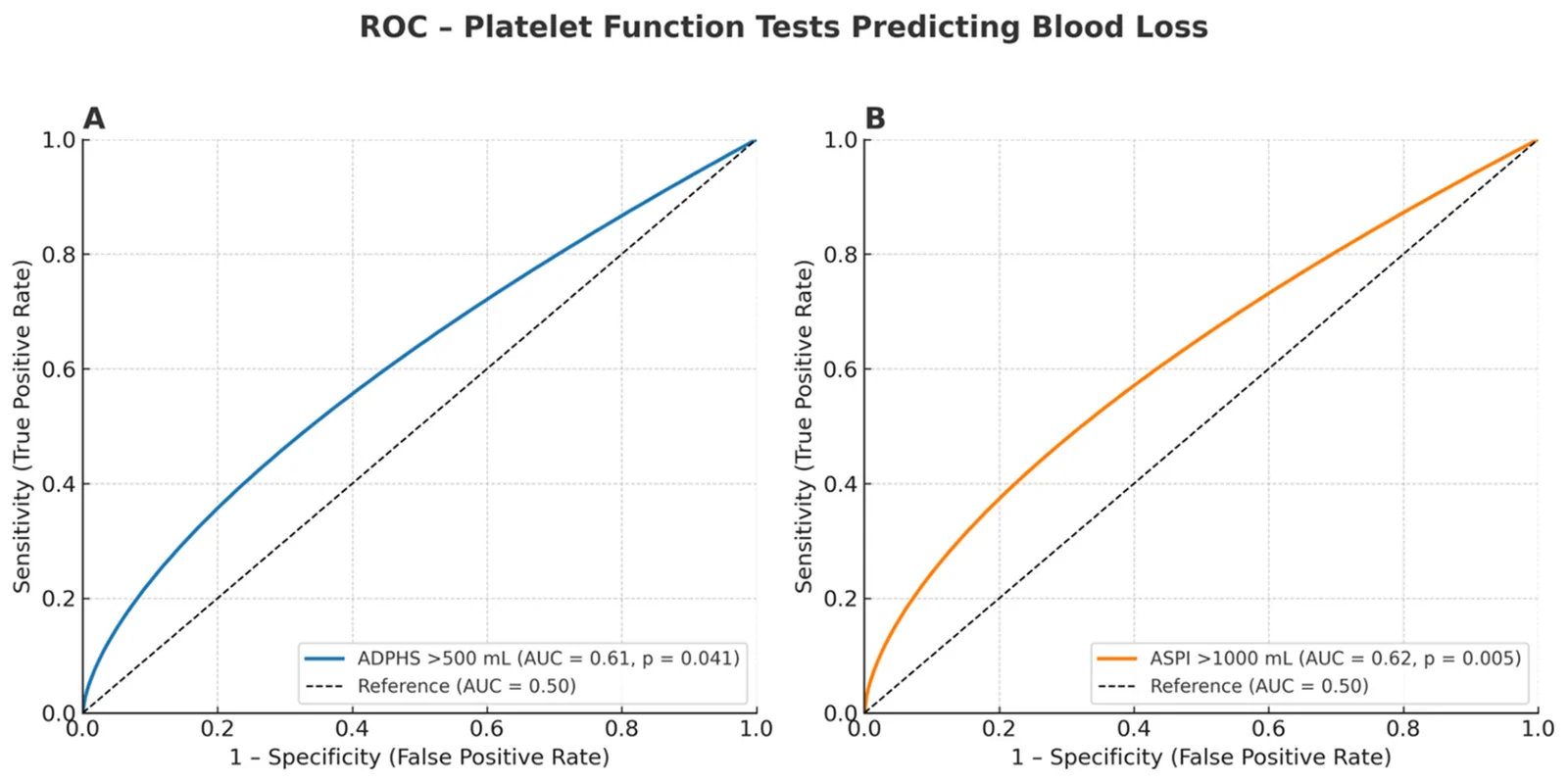
(**A**,**B**) ROC curves of preoperative platelet function tests predicting perioperative blood loss. (**A**) ADPHS as a predictor of total blood loss > 500 mL within the first 24 h. (**B**) ASPI as a predictor of total blood loss > 1000 mL within the first 24 h.

In the ADPHS analysis (Table 3), patients with values ≤ 602.5 (n = 246) had significantly higher transfusion requirements compared with those with values > 602.5 (n = 462). Platelet transfusions were administered in 21.1% vs. 5.1% (*p* < 0.001, χ^2^ = 35.6), while cryoprecipitate was required in 27.6% vs. 17.0% (*p* = 0.001, χ^2^ = 10.7). The overall transfusion rate was also significantly higher in the low-ADPHS group (60.2% vs. 49.2%, *p* = 0.006, χ^2^ = 7.6). By contrast, erythrocyte transfusion (43.9% vs. 40.8%, *p* = 0.433) and plasma administration (7.3% vs. 4.9%, *p* = 0.181) were not significantly different between the groups. In unadjusted ROC analysis, ADPHS showed modest discrimination for total blood loss exceeding 500 mL within the first 24 h (AUC = 0.61, 95% CI 0.51–0.71, *p* = 0.041) (Figure 2B).

For the ASPI test (Table 3), patients with values ≤ 453 (n = 224) showed a trend toward higher transfusion needs but without reaching statistical significance in most categories. The overall transfusion rate was 56.3% vs. 51.2% (*p* = 0.214). Platelet transfusion occurred in 13.8% vs. 9.1% (*p* = 0.056), and erythrocyte transfusion in 45.5% vs. 40.1% (*p* = 0.171). Fresh frozen plasma was administered in 6.7% vs. 5.4% (*p* = 0.483). Cryoprecipitate use was higher in the low-ASPI group (26.3% vs. 18.0%, *p* = 0.011). In unadjusted ROC analysis, ASPI showed modest discrimination for severe postoperative blood loss exceeding 1000 mL within the first 24 h (AUC = 0.62, 95% CI 0.54–0.70, *p* = 0.005), but not for bleeding exceeding 500 mL within the first 24 h or overall transfusion (AUC values 0.47–0.51, *p* > 0.4) (Figure 2A).

To account for baseline differences between the groups, additional multivariable logistic regression analyses were performed with postoperative bleeding > 500 mL within the first 24 h as the dependent variable. The ADPHS model was adjusted for sex, hyperlipidemia, diabetes mellitus, and ejection fraction, whereas the ASPI model was adjusted for age and diabetes mellitus. After adjustment, neither ADPHS ≤ 602.5 AU·min (adjusted OR 1.01, 95% CI 0.74–1.39, *p* = 0.936) nor ASPI ≤ 453 AU·min (adjusted OR 0.89, 95% CI 0.64–1.23, *p* = 0.475) was independently associated with postoperative bleeding > 500 mL within the first 24 h. Absolute 24-h blood loss and blood-component quantities are provided in Table 4. For the reviewer-requested ADPHS absolute-value re-tabulation, raw continuous ADPHS measurements were available for 412 patients; therefore, these descriptive comparisons are reported as available-case analyses without imputation.

### 3.3. Postoperative Viscoelastic Testing

**Figure 3:**
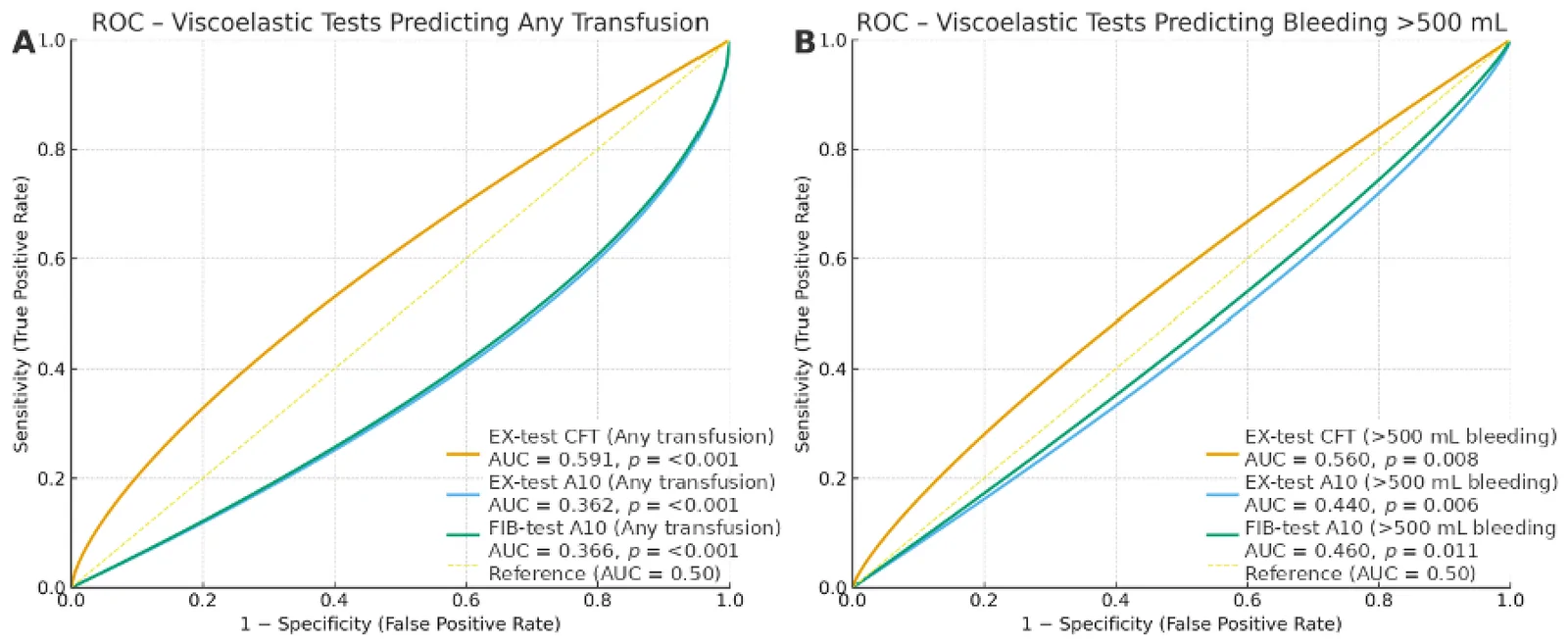
(**A**,**B**) ROC curves for viscoelastic parameters. (**A**) ROC curves of EX-test CFT, EX-test A10, and FIB-test A10 for predicting any transfusion. (**B**) ROC curves of EX-test CFT, EX-test A10, and FIB-test A10 for predicting postoperative bleeding > 500 mL within the first 24 h.

Postoperative viscoelastic testing with ClotPro^®^ showed statistically significant associations with transfusion requirements, although the discriminatory performance of individual parameters was limited. EX-test CFT yielded an AUC of 0.591 (95% CI 0.53–0.65, *p* < 0.001), representing weak discrimination rather than strong predictive performance. EX-test A10 and FIB-test A10 were inversely associated with transfusion requirements, with AUC values of 0.362 (95% CI 0.30–0.43, *p* = 0.001) and 0.366 (95% CI 0.30–0.43, *p* = 0.001), respectively. Because lower A10 values were associated with greater transfusion risk, AUC values below 0.50 reflect the inverse direction of the association and should not be interpreted as clinically useful standalone discrimination (Figure 3A).

For major postoperative bleeding (>1000 mL within the first 24 h), none of the viscoelastic parameters demonstrated useful discriminatory performance (*p* > 0.1). For moderate bleeding (>500 mL within the first 24 h), EX-test CFT (AUC = 0.56, 95% CI 0.48–0.64, *p* = 0.008), EX-test A10 (AUC = 0.44, 95% CI 0.37–0.51, *p* = 0.006), and FIB-test A10 (AUC = 0.46, 95% CI 0.38–0.54, *p* = 0.011) showed statistical associations. However, all AUC values remained close to 0.50, indicating poor discrimination; the values below 0.50 for A10 parameters reflect inverse associations because lower amplitudes were associated with greater bleeding risk (Figure 3B).

### 3.4. Cross-Validation Between Pre- and Postoperative Tests

**Figure 4:**
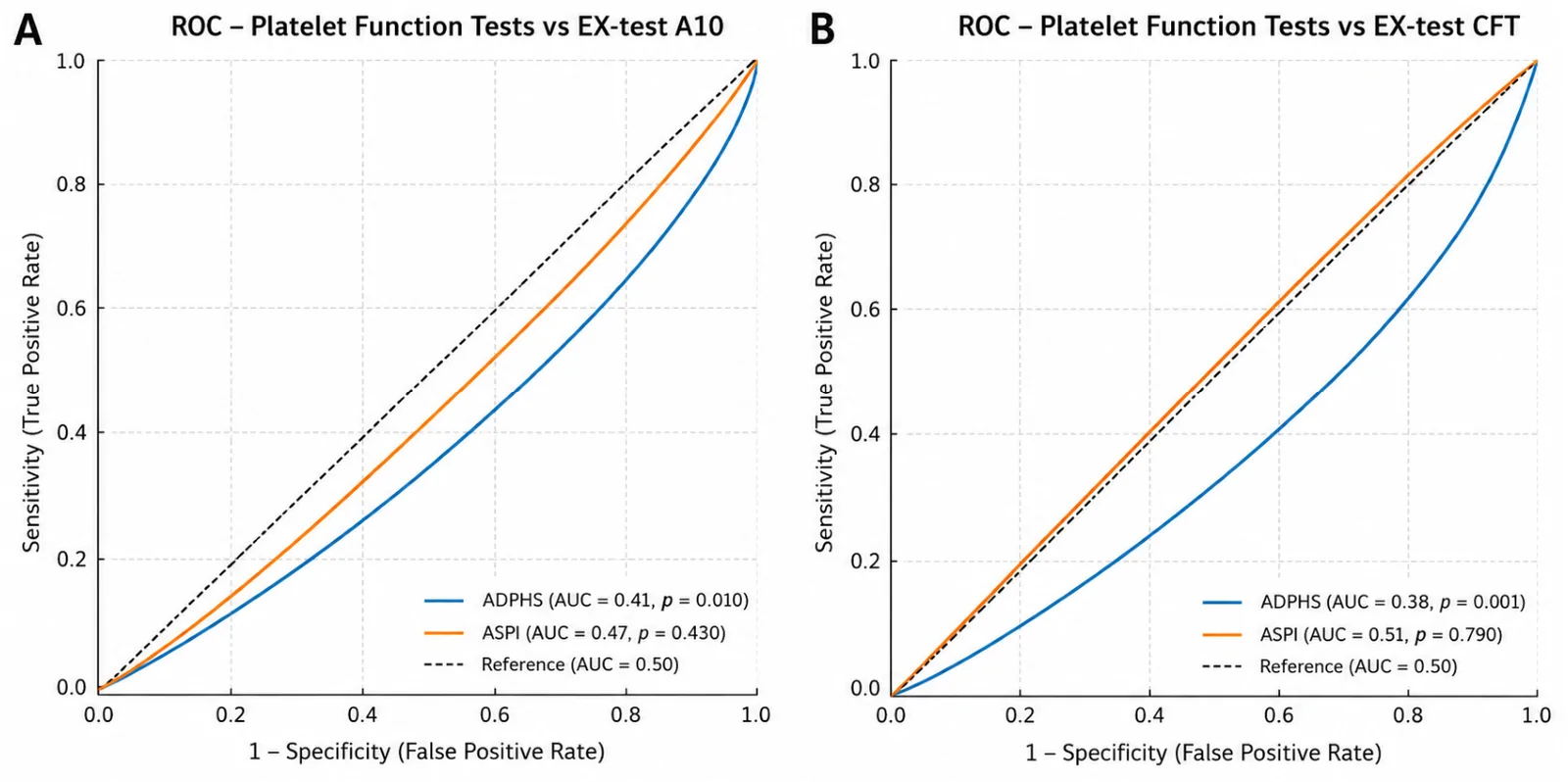
(**A**,**B**) ROC curves of platelet function tests versus viscoelastic parameters in relation to 24-h postoperative blood loss. (**A**) ADPHS and ASPI versus EX-test A10. (**B**) ADPHS and ASPI versus EX-test CFT.

The correlation between preoperative impedance aggregometry and postoperative viscoelastic testing is summarized in Figure 4. In patients classified as high bleeding risk by ADPHS, the ROC analysis relating preoperative platelet function to postoperative EX-test CFT yielded an AUC of 0.38 (*p* = 0.001), while the corresponding ASPI AUC was 0.51 (*p* = 0.79) (Figure 4B). For EX-test A10, the ADPHS AUC was 0.41 (*p* = 0.01), and the ASPI AUC was 0.47 (*p* = 0.43) (Figure 4A). AUC values below 0.50 indicate an inverse direction of association, and because these values remain close to 0.50, they represent limited discrimination rather than strong predictive performance. ASPI values showed no meaningful discriminatory relationship with postoperative viscoelastic parameters.

## 4. Discussion

This study analyzed the relationship between preoperative point-of-care platelet function testing, postoperative viscoelastic testing, bleeding, and transfusion outcomes in patients undergoing isolated on-pump CABG. The main findings were: (i) lower ADPHS values were associated with greater platelet, cryoprecipitate, and overall blood-product use in unadjusted analyses; (ii) lower ASPI values were associated with greater cryoprecipitate use; (iii) ADPHS and ASPI demonstrated only modest discrimination for the prespecified bleeding endpoints; (iv) after adjustment for baseline group differences, neither ADPHS ≤ 602.5 AU·min nor ASPI ≤ 453 AU·min was independently associated with postoperative bleeding > 500 mL/24 h; and (v) postoperative ClotPro^®^ parameters showed statistically significant but clinically limited discriminatory performance. These findings support a cautious interpretation of POC test associations and do not establish independent predictive utility or a transfusion algorithm [10,11,12,13,14,15,16,17,18].

Our findings align with previous reports demonstrating that impaired preoperative platelet aggregation is associated with perioperative bleeding and transfusion after cardiac surgery. In particular, ADP-induced platelet inhibition is clinically relevant in patients exposed to P2Y12 inhibitors such as clopidogrel and ticagrelor. In the present cohort, preoperative aspirin, clopidogrel, and ticagrelor were recorded in 89.3%, 48.0%, and 12.0% of patients, respectively, confirming substantial antiplatelet exposure in the population being studied. Interindividual variability in drug effect and platelet recovery provides an important biological context for interpreting the Multiplate^®^ results. Point-of-care testing may therefore identify residual platelet inhibition that is not fully captured by medication history alone.

The observed association between low ADPHS values and higher transfusion rates supports previous reports indicating that platelet function testing can guide surgical timing and transfusion strategies in cardiac surgery patients. Several studies have shown that reduced ADP-induced platelet aggregation, as measured by Multiplate^®^ or VerifyNow^®^, is predictive of increased postoperative bleeding and transfusion requirements [9,11,15,16,19,20,21,22].

Integrating such testing into perioperative decision-making has been associated with reduced bleeding, optimized transfusion management, and improved patient blood management outcomes. The relatively modest predictive performance of the ASPI test in our study should be interpreted in the context of the high prevalence of preoperative aspirin exposure (89.3%) and the lower bleeding risk generally associated with selective COX-1 inhibition compared with P2Y12 blockade.

The transfusion rates observed in this cohort should also be interpreted in the context of our institutional transfusion practices. Erythrocyte transfusion was considered at hemoglobin <7 g/dL in hemodynamically stable patients and <8 g/dL in hemodynamically unstable patients. In addition, treating clinicians had access to both Multiplate^®^ and ClotPro^®^ results, and these tests were used to inform blood-component therapy. Consequently, transfusion outcomes are susceptible to treatment-incorporation bias: an association between an abnormal POC result and transfusion may partly reflect the clinical response to that result rather than only the underlying biological bleeding risk. This is particularly relevant when interpreting platelet, plasma, and cryoprecipitate transfusion outcomes.

Postoperative viscoelastic testing with ClotPro^®^ provided additional mechanistic insight into coagulation impairment following cardiopulmonary bypass (CPB). The association between prolonged EX-test CFT and increased transfusion requirements is consistent with prior studies demonstrating relationships between viscoelastic parameters, transfusion needs, and bleeding severity after cardiac surgery. Prolonged CFT and reduced EX-test or FIB-test A10 reflect impaired clot propagation and reduced clot firmness, which may occur after CPB. In our cohort, however, the AUC values were generally close to 0.50. Therefore, although several associations reached statistical significance, their discriminatory performance was poor, and they should not be interpreted as strong standalone predictors. None of the viscoelastic parameters demonstrated useful discrimination for major postoperative bleeding (>1000 mL within the first 24 h). Associations observed for moderate bleeding (>500 mL within the first 24 h) should likewise be interpreted in the context of their limited AUC values. These findings favor the use of viscoelastic testing as part of a broader clinical and laboratory assessment rather than as an isolated predictive tool.

The cross-validation analysis revealed that the predictive strength of postoperative ClotPro^®^ parameters was most pronounced in patients with low preoperative ADPHS values, suggesting that those with residual platelet inhibition are particularly vulnerable to the additive effects of CPB-related coagulopathy. Similar interactions between platelet dysfunction and impaired fibrin polymerization—both amplifying bleeding and transfusion risk—have been reported, including studies showing ADP-hypoaggregability predicting bleeding in cardiac surgery and the value of fibrin(ogen)-focused correction when polymerization is deficient [13,23,24,25]. These observations suggest that the two modalities may provide complementary hemostatic information; however, given the low AUC values and observational design, they do not demonstrate incremental clinical benefit from a combined testing strategy.

POC platelet function and viscoelastic tests can characterize different components of perioperative hemostasis, including platelet inhibition and clot-formation abnormalities. Randomized, meta-analytic, and guideline-level evidence shows that viscoelastic-guided algorithms reduce allogeneic blood product exposure, bleeding, and re-exploration, and can shorten ICU stay compared with standard care [26,27,28,29,30]. Our results align with the 2024 EACTS/EACTAIC Guidelines on patient blood management in adult cardiac surgery, which advocates targeted, physiology-driven transfusion strategies and supports incorporating POC platelet and viscoelastic testing into routine cardiac surgical practice [9]. However, the present observational study was not designed to test whether adding preoperative Multiplate^®^ to postoperative ClotPro^®^ improves clinical outcomes or reduces blood-product use compared with a standard transfusion algorithm.

Recent publications further strengthen the contemporary evidence base for perioperative patient blood management. Systematic reviews, meta-analyses, scoping reviews, and guideline-methodology analyses published in 2024–2026 have specifically addressed bleeding-management algorithms in cardiac surgery, viscoelastic-guided hemostatic therapy, desmopressin, intraoperative hemostatic checklists, and pharmacotherapy recommendations for cardiac-surgery PBM [31,32,33,34,35]. These newer data were added to provide a more current context, while older landmark studies were retained when directly relevant to assay methodology or foundational evidence.

From a clinical standpoint, our findings reinforce the role of multimodal point-of-care testing in cardiac surgery as a practical extension of patient blood management (PBM) principles. Integrating preoperative impedance aggregometry with postoperative viscoelastic assays enables more tailored transfusion decisions by identifying the dominant mechanism of bleeding—whether platelet inhibition, fibrinogen depletion, or CPB-induced coagulopathy. Similar approaches have been shown to reduce allogeneic transfusions and re-exploration rates while optimizing hemostatic therapy [26,28,29]. In the current STS/SCA/AmSECT/SABM PBM guideline, viscoelastic testing receives a Class IIa recommendation for guiding transfusion therapy in cardiac surgery, particularly when used in combination with platelet function assays [1]. Our findings should therefore be viewed as hypothesis-generating and supportive of physiology-based assessment rather than as evidence for a new implementable algorithm. Prospective interventional studies are required to establish whether a combined testing strategy provides incremental clinical benefit.

This study has several limitations. First, it was a single-center prospective observational study; therefore, causality and clinical benefit from the testing strategy cannot be inferred. Second, although additional multivariable analyses adjusted for baseline variables that differed significantly between the ADPHS and ASPI groups, the adjusted associations with bleeding > 500 mL were not significant, and residual confounding remains possible. Potential bleeding determinants not included in the final models, including renal or liver dysfunction, baseline hemoglobin, platelet count, and routine coagulation parameters, may influence outcomes and should be incorporated in future externally validated models. Third, POC test results were visible to treating clinicians and were used to guide blood-component therapy; therefore, transfusion endpoints are susceptible to treatment-incorporation bias. Fourth, component therapy was individualized according to POC findings and clinical assessment rather than a single fixed numerical algorithm, which may limit reproducibility across centers. Fifth, testing was limited to predefined perioperative time points, and serial postoperative measurements might have captured additional coagulopathic events. Finally, the cost-effectiveness and incremental clinical utility of a combined Multiplate^®^ and ClotPro^®^ approach were not assessed. Multicenter prospective interventional studies are needed before routine implementation can be recommended. In addition, although perioperative antithrombotic exposure is now reported, the analysis did not model drug dose, the exact timing of the last preoperative dose, adherence, or the timing of postoperative re-initiation; these factors may influence platelet function and viscoelastic results.

## 5. Conclusions

Preoperative Multiplate^®^ results and postoperative ClotPro^®^ parameters were associated with bleeding and transfusion outcomes in several unadjusted analyses, but their discriminatory performance was modest. After adjustment for baseline group differences, neither ADPHS ≤ 602.5 AU·min nor ASPI ≤ 453 AU·min was independently associated with postoperative bleeding > 500 mL/24 h. In addition, transfusion outcomes must be interpreted in the context of unblinded POC-guided component therapy. Accordingly, the present study supports the use of platelet function and viscoelastic testing as complementary sources of hemostatic information, but it does not establish a standalone prediction tool, a universal cutoff, or an implementable combined algorithm. Further prospective interventional validation is required to determine whether combined testing improves patient blood management outcomes.

## Figures and Tables

**Table 3:** Association between platelet function test thresholds and perioperative transfusion requirements.

	ADPHS ≤ 602.5 (n = 246)	ADPHS > 602.5 (n = 462)	*p*-Value	ASPI ≤ 453 (n = 224)	ASPI > 453 (n = 484)	*p*-Value
**Erythrocyte** **transfusion**	108 (43.9%)	185 (40.8%)	0.433	102 (45.5%)	194 (40.1%)	0.171
**Platelet** **transfusion**	52 (21.1%)	23 (5.1%)	<0.001	31 (13.8%)	44 (9.1%)	0.056
**Fresh** **frozen plasma**	18 (7.3%)	22 (4.9%)	0.181	15 (6.7%)	26 (5.4%)	0.483
**Cryoprecipitate**	68 (27.6%)	77 (17.0%)	0.001	59 (26.3%)	87 (18.0%)	0.011
**Any** **transfusion**	148 (60.2%)	223 (49.2%)	0.006	126 (56.3%)	248 (51.2%)	0.214

**Table 4:** Absolute 24-h postoperative blood loss and blood-component quantities according to platelet function strata. Data are median [IQR] with total quantity shown after the semicolon.

Outcome	ADPHS ≤ 602.5 (Available n = 229)	ADPHS > 602.5 (Available n = 183)	*p*-Value	ASPI ≤ 453 (n = 224)	ASPI > 453 (n = 484)	*p*-Value
24-h chest-tube drainage, mL	600 [420–830]; total 170,889	555 [420–750]; total 125,663	0.191	580 [430–852]; total 157,965	586 [410–800]; total 336,214	0.779
RBC units per patient	0 [0–1]; total 202	0 [0–1]; total 161	0.705	0 [0–2]; total 235	0 [0–1]; total 436	0.142
Platelet units per patient	0 [0–0]; total 218	0 [0–0]; total 77	0.012	0 [0–0]; total 253	0 [0–0]; total 349	0.053
FFP units per patient	0 [0–0]; total 24	0 [0–0]; total 17	0.814	0 [0–0]; total 35	0 [0–0]; total 67	0.501
Cryoprecipitate units per patient	0 [0–0]; total 643	0 [0–0]; total 355	0.070	0 [0–6]; total 649	0 [0–0]; total 1105	0.022

Note: ADPHS absolute-value analyses use available raw continuous ADPHS measurements (n = 412; 229 ≤ 602.5 and 183 > 602.5) and were performed without imputation. ASPI values use the corrected full-cohort grouping (224 ≤ 453 and 484 > 453).

## Data Availability

The original contributions presented in this study are included in the article. Further inquiries can be directed to the corresponding author.

## Abbreviations

The following abbreviations are used in this manuscript:

Abbreviation	Definition
ACS	Acute Coronary Syndrome
ADPHS	Adenosine Diphosphate High-Sensitivity Test (Multiplate^®^ ADP test)
ASPI	Arachidonic Acid-Induced Platelet Aggregation Test (Multiplate^®^ ASPI test)
A10	Amplitude at 10 min
AUC	Area Under the Receiver Operating Characteristic Curve
CABG	Coronary Artery Bypass Grafting
CFT	Clot Formation Time
CI	Confidence Interval
ClotPro^®^	ClotPro Viscoelastic Hemostatic Analyzer
COPD	Chronic Obstructive Pulmonary Disease
CPB	Cardiopulmonary Bypass
EACTAIC	European Association of Cardiothoracic Anaesthesiology and Intensive Care
EACTS	European Association for Cardio-Thoracic Surgery
EBCP	European Board of Cardiovascular Perfusion
EX-test A10	Amplitude at 10 min in the ClotPro^®^ EX-test
EX-test CFT	Clot formation time in the ClotPro^®^ EX-test
EX-test	ClotPro^®^ extrinsic pathway viscoelastic assay
FIB-test	ClotPro^®^ fibrinogen-contribution viscoelastic assay
HR	Hazard Ratio
IBM SPSS	IBM Statistical Package for the Social Sciences
ICU	Intensive Care Unit
IRB	Institutional Review Board
MAC	Minimum Alveolar Concentration
MI	Myocardial Infarction
MICS	Minimally Invasive Cardiac Surgery
PBM	Patient Blood Management
PCI	Percutaneous Coronary Intervention
POC	Point-of-Care
P2Y12	Platelet Adenosine Diphosphate (ADP) Receptor P2Y12
Q-Q	Quantile–Quantile Plot
ROC	Receiver Operating Characteristic
ROTEM^®^	Rotational Thromboelastometry
SD	Standard Deviation
STS	Society of Thoracic Surgeons
TEG^®^	Thromboelastography
TIA	Transient Ischemic Attack
